# Well-differentiated liposarcoma disguised as a recurrent lipoma of the forearm flexor compartment: A case report

**DOI:** 10.1016/j.ijscr.2020.05.063

**Published:** 2020-05-30

**Authors:** Wahyu Widodo, Wildan Latief, Dina Aprilya

**Affiliations:** Department of Orthopaedic and Traumatology, Faculty of Medicine Universitas Indonesia, Cipto Mangunkusumo Hospital, Indonesia

**Keywords:** Giant lipoma, Forearm, Well differentiated liposarcoma, Lipoma-like

## Abstract

**Introduction:**

Lipomas are common benign mesenchymal tumor that arise from the adipose tissue. Differential diagnosis between the benign and malignant form (liposarcoma) must be made to prevent complication and recurrence.

**Presentation of case:**

We reported a 63-year-old man who presented with recurrent large-sized mass on the right forearm with the first presentation approximately 28 years before being treated in our center. Main complaints were discomfort, social embracement, and difficulty in dressing. There were no compression symptoms observed. We performed marginal excision and sent the sample for histopathological examination. The work has been reported in line with the Surgical Case Report (SCARE) criteria.

**Discussion:**

During one year of post-operative period, no recurrence was detected and a complete relief of symptoms was as expected.

**Conclusion:**

Diagnostic challenge in such a large-sized (giant) lipoma is in differentiating it with the malignant form. Surgical excision is the choice of treatment. However, long-term follow up is needed due to the risk of recurrence.

## Highlights

• The giant size and other unusual characteristics raised the awareness of malignant lipomatous mass.
• Differential diagnosis between giant lipoma and liposarcoma is of great importance.
• Post-operative histopathological diagnosis was well-differentiated liposarcoma lipoma-like.

## 1 Introduction

Lipoma is one of the most common benign soft tissue tumors. It commonly occurs subcutaneously, however its appearance on any other plane has been reported. Large-sized lipoma or ‘giant lipoma’ is considered if the size is greater than 5 cm in diameter. We presented a case of a 63-year-old male with giant lipoma on the flexor compartment of his right forearm which was treated with complete removal of the lipomatous mass with marginal excision. Post-operative histopathological diagnosis was well-differentiated liposarcoma lipoma-like. No recurrence had been observed on the 1-year follow up. Our work has been reported in line with the Surgical Case Report (SCARE) Guidelines [1].

## 2 Case

**Fig. 1:**
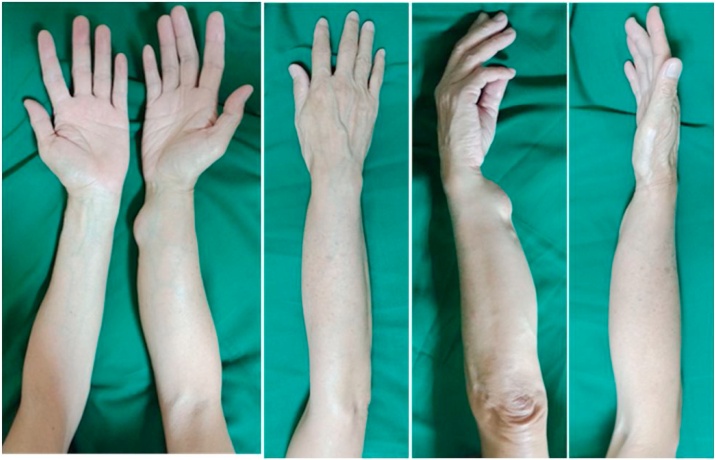
Lump on the volar side of right forearm.

A 63-year old male was referred to orthopedic outpatient clinic at our institute with a large lump on the right forearm region. The lump initially appeared 28 years ago and had re-appeared for three times after three times removal. The latest removal was 7 years before admitted to our center and the latest recurrence was in the next 2 years. The patient was only complaining about mild discomfort and difficulty in wearing clothes. The non-tender lump was increasing in size slowly from the size of quail eggs with well-defined border around the wrist until it occupied the whole flexor compartment of the right forearm. The right forearm circumference was 20 cm compared to 7 cm on the contralateral (Fig. 1). There was neither neurological deficit nor signs of compartment syndrome distal to the lump.

The laboratory markers showed no abnormality except for high blood glucose since the patient has diabetes mellitus since the past 11 years. He also had a hyperlipidemia with increase of both total and LDL cholesterol level.

**Fig. 2:**
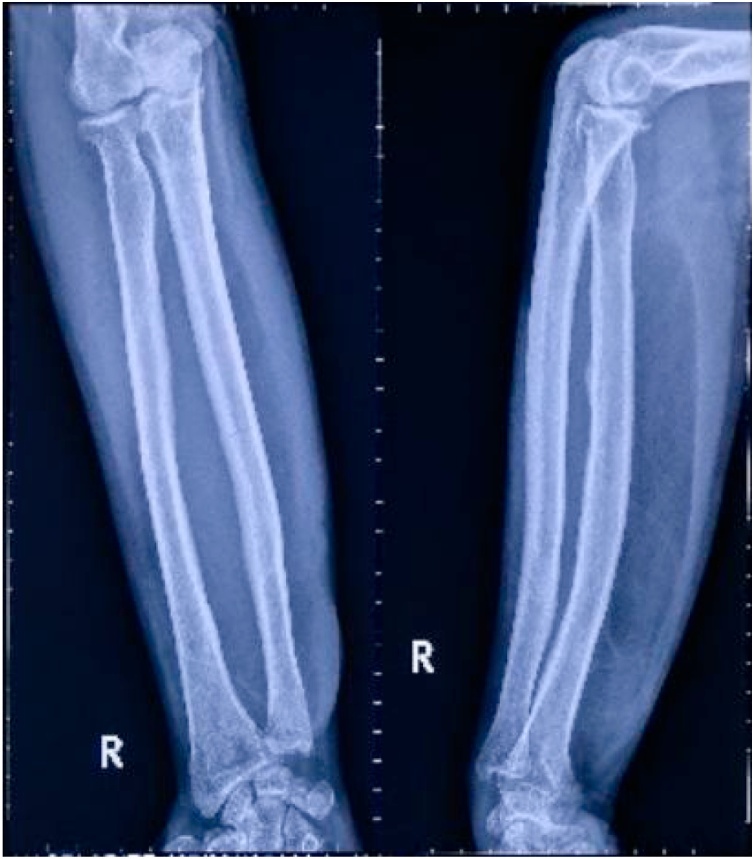
A more radiolucent soft tissue mass compared to surroundings on the volar part of right forearm on the level of proximal shaft radius-ulna to metaphyseal region of distal radius-ulna. Cortical thickening was noted on mid-shaft radius. No bony destruction or lesions were observed.

**Fig. 3:**
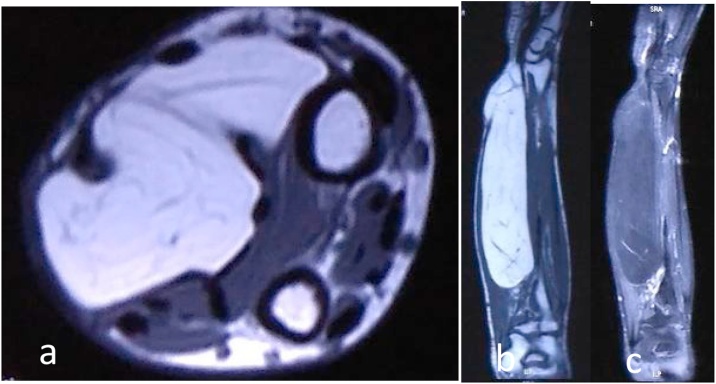
Well-defined margin mass lies inter-muscularly deep into the flexor carpi radialis (FCR) muscle and common digital flexor with the size of 20.2 × 5.1 × 3.6 cm (a). Surrounding structures were pressed without any infiltrations observed. On T1W sequence this fatty dominance lesion has hyperintensity (b) which became hypointense on STIR sequences (c). There were simple septations within the mass which showed contrast enhancement.

Patient underwent radiograph assessment with plain x ray which suggest lipomatous mass (Fig. 2) and Magnetic Resonance Imaging (MRI) which concluded a prominent lipomatous mass on intermuscular region with possible benign lesion or low-grade malignancy (Fig. 3). On the Clinico-Pathological Conference (CPC), this more likely to be benign lipomatous lesion and was decided to undergo surgical exploration, marginal excision and biopsy.

**Fig. 4:**
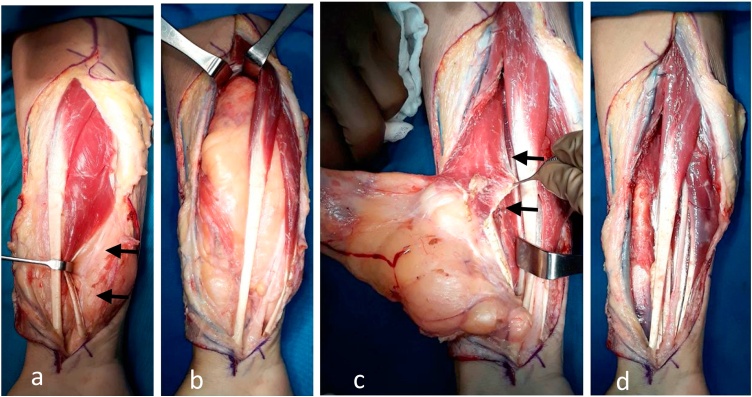
Yellowish lipomatous mass with pseudo-capsule. (a) The mass was subcutaneously located on the wrist region with proximity to the median n. (arrow) and (b) became intermuscular as it extended deep into the more proximal location on the forearm and occupied the space between FCR and FDC muscle. (c) The tumor was detached from FPL (arrow) as its deepest adjacent structure. (d) Final clinical picture after tumor removal.

Surgical exploration was therefore performed under general anesthesia and with the application of a hemostatic tourniquet. A lazy-S design was made approximately one cm lateral to the antecubital fossa and extended distally until distal wrist crease. The incision started on the ulnar-volar side on the distal forearm where the initial mass appeared and where it most superficially located under the skin. After skin incision and entering the subcutaneous plane on the wrist region, there was a lipoma-like lesion which continued sub-muscularly underneath the FDS muscle. Blunt dissection through muscle fiber confirmed a deep-seated lipoma lying over the Flexor Pollicis Longus (FPL) muscle. Running through the lipoma was a shiny cord-like structure, which was identified as the median nerve. The mass was carefully dissected off the median n. until releasing the transverse carpal ligament to ensure that the nerve was left intact. The mass was found to compress the surroundings; however, it was relatively easy to remove due to the pseudo-capsule and the intermuscular location (Fig. 4).

**Fig. 5:**
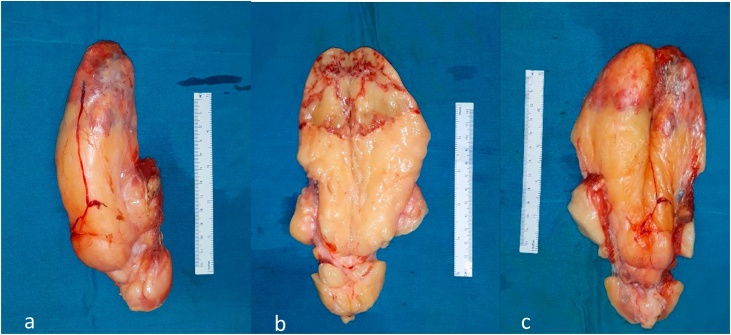
Lobulated, well circumscribed, well-vascularized and rubbery yellowish mass with a firmer area on its superior pole. There were no fat necrosis or hemorrhage. (a) before divided, (b) inner side, and (c) outer side.

The 15 × 8 × 5.5 cms mass was sent for histopathological analysis. The mass was well-vascularized as observed that there were big vessels that gave blood supply to the mass and these vessels infiltrated the mass especially at the superior pole, where it gave a yellow-red appearance on that region. The mass consistency was generally rubbery with a firmer consistency at the superior pole. (Fig. 5).

**Fig. 6:**
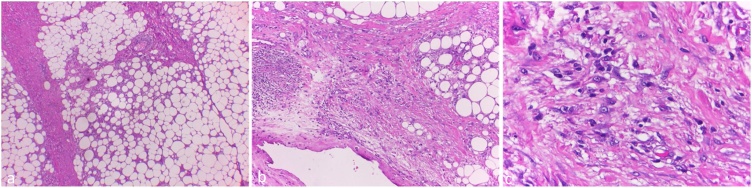
Histopathological picture in HE staining with various magnification. There was adipose cell proliferation with varying size and scattered atypical cells, of which, some has nucleoli. (a) 40×, (b) 100×, and (c) 400× magnification.

Histopathological analysis suggested a lipomatous tumor, low grade, most suitable with well-differentiated liposarcoma (WDL)-lipoma like subtype (Fig. 6).

**Fig. 7:**
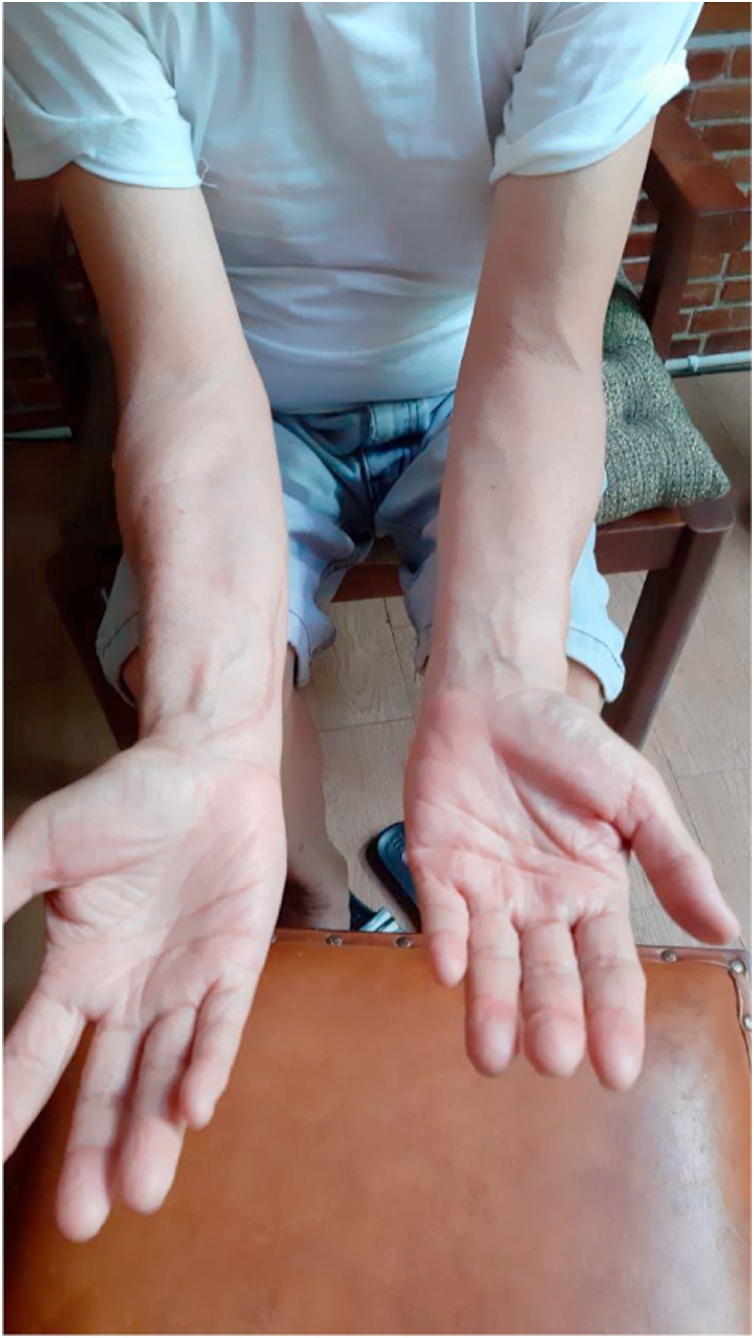
Clinical appearance at one year follow up.

During routine follow-up in the outpatient clinic at one and three months, the wound had healed well and there were no neurological deficit, functional impairment or recurrence of the lump observed. At one-year follow-up the scar healed nicely without recurrence of the mass (Fig. 7).

## 3 Discussion

The etiology and pathogenesis of lipomas remain unknown, although genetic endocrine and traumatic factors have been suggested. Our patient had diabetes mellitus in the past 11 years which was treated by oral anti-diabetic drugs. He also had a long-standing hyperlipidemia with the increase of total cholesterol and LDL cholesterol level.

Typical lipoma occurs more often in young adults, women (thigh), and in overweight populations. They vary in size [2]; when the size is greater than 5 cm in diameter, the term ‘giant lipoma’ is considered, as it may raise the awareness to other malignant form of lipomatous mass such as liposarcoma [3]. Our patient is a 63-year-old normal-weight man which had first presentation of the lipoma approximately 28 years ago; this is an uncommon characteristic for the typical lipoma. Furthermore, its presentation in volar part of the forearm is also unusual from those of giant lipomas which occur most frequently in cervical region, thorax, and lower limbs (inguinal region and thigh) [4].

Although the definitive diagnosis of giant lipoma can be made only by histopathological examination, the differential diagnosis between giant lipoma and liposarcoma is of great importance. Once suspected, other investigations must be conducted to provide more information about the tumor such as ultrasonography (USG), computed tomography (CT) scan and magnetic resonance imaging (MRI) as well as technetium-99 diethylenetriaminepentaacetic acid scanning [2,3,5].

In our case, the plain radiograph showed a radiolucent soft tissue mass along the shaft of radius ulna on the volar part of the right forearm. The MRI showed homogeneous hyperintense fat signal on T1-weighted MR images whereas T2-weighted images showed hypointense fat-suppression signal. The mass appeared inter-muscularly between FCR and FDC with well-defined margin and no infiltration to surrounding structures, raising the susceptibility of a lipomatous tumor with benign characteristics.

Surgical is the mainstay treatment for giant lipoma due to the size, recurrence risk and potential malignant transformation. Complete surgical resection, with preferably wide-margin, is recommended to prevent dedifferentiation and recurrence. Adjuvant radiation therapy may benefit in large high-grade liposarcoma and in any other liposarcoma when the wide resection cannot be obtained. The value of chemotherapy is still debatable as adjuvant to surgical therapy [6].

In our case due to the complicated structure and relatively narrow space at the forearm, we could not perform wide resection with much negative margins. Intraoperatively, we found that the mass was deep-seated, well-vascularized, well-circumscribed, lobulated and had a rubbery consistency. Those characteristics were suitable for the lipomatous mass but not specifically present liposarcoma. The cut surface was yellow with more hyperemic areas on the superior pole of the mass. A pseudo-capsule which was formed by the continuous pressure on surrounding tissue made the dissection and enucleation of the mass uncomplicated. Since the mass was intermuscular, we were able to remove the tumor without affecting the adjacent muscular tissue while preserving the neurovascular bundle, in this case, the median nerve particularly [3,4,7].

Post-operative histopathological exam revealed this giant lipomatous mass as the well differentiated liposarcomas (WDL) lipoma-like subtype. Account for 40–45 % of liposarcomas, WDL usually diagnosed at the 5th decades of life with slight male predominance. WDL are locally aggressive mesenchymal tumors composed of mature adipocytes and stromal cells with at least focal cytologic atypia. WDL are considered as low grade lipogenic tumors with morphologic subtypes dependent on the adipocytic component and background cellularization characteristic. Several histologic subtypes have been described: lipoma-like, sclerosing, inflammatory, mixed, lipo-leiomyosarcoma, WDL with low grade osteosarcoma-like and spindle cell subtype. Lipoma-like subtype, being the most common form of WDL, is grossly indistinguishable from lipoma. It frequently contains lipoblast and scattered atypical cells may be diffuse or extremely rare [[7], [8], [9]].

In this report, surgical excision resulted in complete relief of symptoms and significant esthetic also became evident in the affected forearm. There was no recurrence in the 1-year follow up. However, we still closely observe this patient for any recurrence since the possible risk of recurrence in the WDL as well as inter-muscularly located lipomatous mass.

## 4 Conclusion

The slow growing nature and radiological characteristic of lipoma brought confusion with the benign lesion. However, the giant size and other unusual characteristics (gender, age, mass location and recurrences) raised the awareness of malignant lipomatous mass. Appropriate diagnosis and complete surgical resection of this tumor provided symptoms relief and esthetic improvement on the affected limb. Long-term evaluation must be conducted in order to detect recurrence. We plan for the wide excision followed by radiotherapy for possible recurrence mass in the future.

## Declaration of Competing Interest

The authors declare no conflicts of interest.

## Funding

The authors report no external source of funding during the writing of this article.

## Ethical approval

Ethical approval was not required in the treatment of the patient in this report.

## Consent

Written consent has been received from the subject.

## Author contribution

Dina Aprilya contributes to the study concept or design, data collection and writing the paper.

Wildan Latief contributes in the study concept or design, data collection, analysis and interpretation, oversight and leadership responsibility for the research activity planning and execution, including mentorship external to the core team.

Wahyu Widodo contributes in the study concept or design, data collection, analysis and interpretation, oversight and leadership responsibility for the research activity planning and execution, including mentorship external to the core team.

## Registration of research studies

Not required.

## Guarantor

Wahyu Widodo is the sole guarantor of this submitted article.

## Provenance and peer review

Editorially reviewed, not externally peer-reviewed.
